# Complete genome sequence of *Lactiplantibacillus plantarum* BBC32B isolated from human feces sample

**DOI:** 10.1128/MRA.00645-23

**Published:** 2023-10-11

**Authors:** Vikram Kumar, Neetu Kumra Taneja, Shakti Kumar, Atul Vashist, Jayesh J. Ahire, Vijendra Mishra

**Affiliations:** 1 Department of Basic and Applied Sciences, NIFTEM, Sonepat, Haryana, India; 2 Centre for Advanced Translational Research in Food Nanobiotechnology (CATR-FNB), NIFTEM, Sonepat, Haryana, India; 3 Department of Molecular Medicine and Biotechnology, Sanjay Gandhi Postgraduate Institute Medicine Sciences, Lucknow, Uttar Pradesh, India; 4 Department of Biotechnology, School of Engineering and Applied Sciences, Bennett University, Greater Noida, Uttar Pradesh, India; 5 Kwbiotics, Nashik, Maharashtra, India; University of Rochester School of Medicine and Dentistry, Rochester, New York, USA

**Keywords:** *Lactiplantibacillus plantarum*, genome, riboflavin

## Abstract

We report complete genome sequence of *Lactiplantibacillus plantarum* BBC32B, which was isolated from human feces sample and submitted to Microbial-Type Culture Collection (MTCC), India with deposition number MTCC 25432. The bacteria from Lactobacillaceae family contained 3,411,152 bp; 3,425 protein coding genes, sharing 69.67% average nucleotide identity with closest species of *Lactobacillus brevis* ATCC367.

## ANNOUNCEMENT

This study describes the whole-genome sequencing (WGS) of a riboflavin-producing lactic acid bacteria isolated from human feces (1). The riboflavin-producing strain BBC32B was submitted to Microbial Type Culture Collection and Gene Bank (MTCC) Chandigarh, India (https://www.imtech.res.in/facilities/microbial-type-culture-collection---mtcc) for identification of authentic microbial culture and species.

Prior to culturing the strain (*Lactiplantibacillus plantarum* BBC32B/MTCC25432) for DNA isolation, it underwent three sub-culturing steps from the original cryo-stock in deMan Rogosa Sharpe (MRS) broth. Each sub-culturing step was followed by overnight incubation at 37°C. Subsequently, for genomic DNA isolation, a 1% inoculum of the culture was introduced into MRS broth and incubated at 37°C for 24 h (2). The genomic DNA of the strain was extracted by Qiagen Genomic DNA Purification Kit as per the instruction given by the manufacturer. Purity of genomic DNA and yield was checked by nanodrop (DS-11 + spectrophotometer) and confirmed by measuring the OD260/OD280 ratio lying between 1.8 and 2.0, thereby indicating no significant RNA or protein contamination. The isolated DNA was stored at −20°C till further analysis. The verified and pure genomic DNA was selected for WGS by Illumina HiSeq 2500 (Illumina, Inc., USA) high-throughput sequencing platform. Approximately, 100 ng of genomic DNA was used for library preparation (3). The Nextera XT DNA Library Preparation Kit (Illumina, Inc., USA) was used for pair-end sequencing and sample indexing. Quantification of DNA library was performed by Qubit Flurometer using Qubit dsDNA HS Assay Kit (Invitrogen), and amplicon length was checked using 2100 Bioanalyzer instrument. The final libraries were pooled and sequenced using HiSeq2500 platform following 2 × 250 paired-end chemistry (4).

The generated sequence was in fastq format and preprocessed by read quality assessment by FastQC (https://www.bioinformatics.babraham.ac.uk/projects/fastqc/) program. Based on the quality report of fastq files, a total number of 4,406,230 reads were generated. The raw reads were trimmed by Trimmomatic (v.39) tool (http://www.usadellab.org/cms/?page=trimmomatic) and used to retain adaptor free high-quality sequences based on phred score (Q ≥20) for further analysis (5). The adapter trimming was performed using fastq mcf (v- 1.04.803) (https://hbctraining.github.io/Intro-to-rnaseq-hpc-salmon/lessons/qc_fastqc_assessment.html). The adapter trimmed reads were aligned to the reference genomes of bacteria, virus, and archaea to check the alignment percentage using Burrows-Wheeler Aligner (BWA) (0.7.12). To eliminate any human genome contamination in the sample, we aligned the pre-processed reads to the human genome (hg19) using the Burrows-Wheeler Aligner-Maximal Exact Match (BWA-MEM) aligner. Unexpected contamination may arise during DNA extraction, from handler’s skin or personnel protective equipment, or cross-contamination from surrounding.

The uncontaminated sequences were then taken for further alignment with known bacterial, fungal, viral, and archaea genomes using BWA-MEM aligner. Around 1.3% of the reads map to the human genome, and 97% to the bacterial genome.

The adapter-trimmed reads were aligned to the nearest reference genome of species *Lactiplantibacillus plantarum* (*Lactobacillus plantarum*), which was selected based on Blast results and their reference fasta files were taken from NCBI (https://blast.ncbi.nlm.nih.gov/Blast.cgi). Then, the consensus was generated using the samtools. Furthermore, the coverage and depth statistics were generated using the bedtools version 2.30.0 and in-house perl script. The bedtools is a versatile toolset for genomic DNA analysis that offers a wide range of functionalities for comparing and manipulating genomic features. Perl scripts can facilitate the analysis of nucleotide sequences. Table 1 summarizes additional genetic data obtained using Prokka annotation results.

**TABLE 1:** Genomic characteristics of *Lactiplantibacillus plantarum* BBC32B/MTCC 25432 obtained using Prokka annotation results^*a*^

Features	Findings for the strain
Genome	Undefined (Taxonomy ID: 1578)
Domain	Bacteria
Taxonomy	Bacteria; Terrabacteria group; Bacillota; Bacilli; Lactobacillales; Lactobacillaceae; undefined undefined undefined
Total sequence length (bp)	3,411,152
GC content (%)	44.3
Contig *N*_50_ (bp)	420,065
Contig *L*_50_ (bp)	3
Number of contigs (with PEGs)	77
Number of subsystems	240
Number of CDSs	3,425
Number of RNAs	78

^*a*^ CDSs, coding DNA sequences; PEGs, protein-encoding genes; GC, guanine, cytosine.

## Data Availability

Online data are available at https://www.ncbi.nlm.nih.gov/bioproject/PRJNA922370/. The complete genome sequence and annotation data for strain Lactiplantibacillus plantarum BBC32B/MTCC 25432 were deposited in GenBank under accession number SRR23032923. The associated BioProject and BioSample accession numbers are PRJNA922370 and SAMN32652500, respectively.
